# Ocrelizumab-assoziierte schwere Neutropenie: eine unterschätzte Komplikation der Therapie mit CD20-Antikörpern bei Multipler Sklerose?

**DOI:** 10.1007/s00115-023-01507-2

**Published:** 2023-06-09

**Authors:** Felix Hess, Paula Uibel, Achim Berthele, Bernhard Hemmer

**Affiliations:** Klinik und Poliklinik für Neurologie, Klinikum rechts der Isar der Technischen Universität München, München, Deutschland

## Fall 1

Eine 39-jährige Patientin stellte sich im Juli 2022 notfallmäßig in unserer Klinik vor. Aufnahmegrund war eine Verschlechterung des Allgemeinzustandes mit Gliederschmerzen sowie Aggravation der vorbestehenden Gangstörung bei spastischer Paraparese im Rahmen ihrer bekannten initial schubförmig verlaufenden Multiplen Sklerose (MS).

Unter den seit der Erstdiagnose im Jahr 2000 zwischenzeitlich applizierten immunmodulatorischen Therapien mit Interferon-β-1a (Interferon-β-1a s.c. 2001–2003, Interferon-β-1a i.m. 2009–2011) sowie Dimethylfumarat (2014–2018) traten jeweils Schübe auf, unter Natalizumab (2011–2012) hatte sich der Verlauf zwar zwischenzeitlich stabilisiert, jedoch wurde die Therapie bei JC-Virus-Serokonversion im November 2012 beendet. Seit April 2018 wurde die Patientin mit Ocrelizumab (OCR) behandelt, was aufgrund ihres Untergewichts (BMI < 17) seit Mai 2019 in reduzierter Dosis (300 mg alle 6 Monate, off-label) verabreicht wurde. Von November 2019 bis Mai 2021 wurde die Therapie in Schwangerschaft und Stillzeit zwischenzeitlich pausiert. Unter OCR stellte sich bildgebend ein stabiler Befund ein, klinisch traten seither keine Schübe mehr auf (EDSS 3,5 Punkte). Die letzte OCR-Gabe war im Mai 2022 erfolgt.

Im Rahmen der aktuellen Vorstellung, 66 Tage nach der letzten OCR-Gabe, imponierte wie vorbekannt eine spastische Paraparese mit insbesondere Affektion der Fußhebung links (MRC 3–4/5). Die Patientin bot mit 38,8 °C febrile Temperaturen und laborchemisch eine Infektkonstellation mit einer in erster Linie schweren Neutrozytopenie von 47 Zellen/µl sowie einer Grad-3-Lymphopenie (Leukozyten 930/µl, Lymphozyten 186/µl, B‑Zellen 0/µl, CRP 19,8 mg/dl, PCT 1,6 ng/ml). In einer der Blutkulturen war *Staphylococcus warneri* nachweisbar, differenzialdiagnostisch erscheint allerdings eine Kontamination möglich. Klinisch sowie in der Umfelddiagnostik fand sich kein eindeutiger Infektfokus.

**Figure 1:**
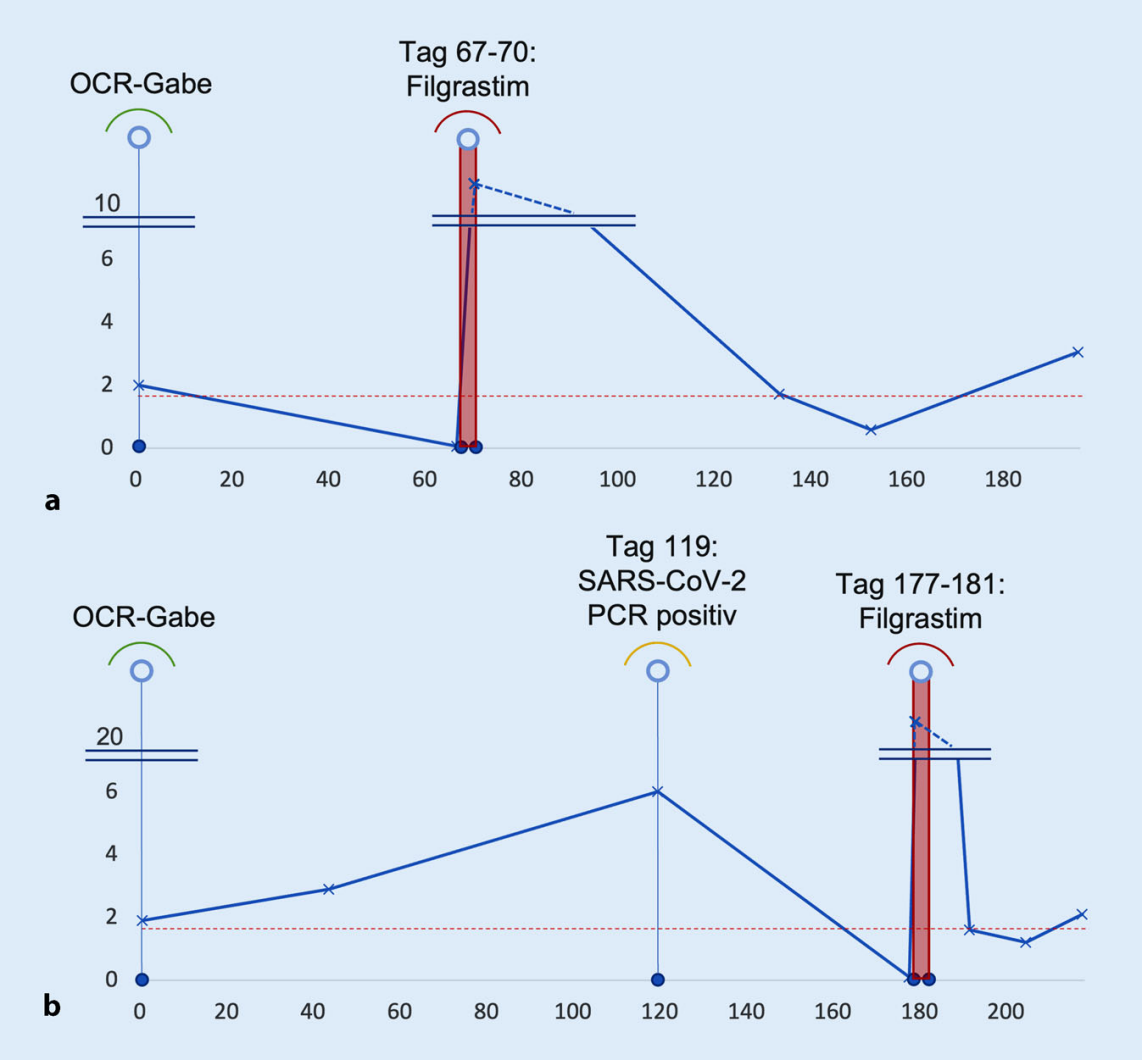

Die Patientin wurde stationär aufgenommen und für 7 Tage kalkuliert breitbandantibiotisch behandelt. Es erfolgte außerdem eine Therapie mit Filgrastim für 4 Tage. In der Folge kam es sehr rasch zu einer anhaltenden Normalisierung der neutrophilen Granulozytenzahl (Abb. 1) sowie zu einer deutlichen Besserung des klinischen Zustandes. Die Therapie mit OCR wurde beendet. In Anbetracht des Nutzen-Risiko-Profils ist eine Anschlusstherapie mit Natalizumab ab Januar 2023 unter engmaschigen Kontrollen der JCV-Serologie sowie MRT-Kontrollen geplant.

## Fall 2

Ein 38-jähriger Patient stellte sich im November 2022 elektiv zur Verlaufskontrolle bei bekannter schubförmig verlaufender MS unter Therapie mit OCR vor.

Vorgeschichtlich war nach Erstmanifestation mit jeweils myelitischen Schüben im Mai und Juli 2015, symptomatisch durch sensible Defizite von Rumpf und unteren Extremitäten, eine Therapie mit Dimethylfumarat begonnen worden. Hierunter kam es im April 2020 zu einem weiteren Schub (sensible Defizite des Rumpfes, sensomotorisches Defizit des rechten Beines) mit magnetresonanztomographischem Nachweis dreier neuer myelitischer Läsionen. Die Symptomatik zeigte sich im Verlauf auch ohne Schubtherapie vollständig regredient, die immunmodulatorische Therapie wurde im Juli 2020 auf OCR umgestellt, worunter sich ein klinisch wie bildgebend stabiler Verlauf einstellte. Seit der Erstgabe im Jahr 2020 liegen Laborkontrollen mit Differenzialblutbild jeweils im Zeitraum von 6 bis 12 Wochen nach OCR-Infusion vor, in denen sich Leukozyten sowie neutrophile Granulozyten (1800–2900/µl) stets normwertig gezeigt hatten. Die letzte Gabe war im Mai 2022 erfolgt. Im September kam es zu einer oligosymptomatischen COVID-19-Erkrankung (PCR positiv), von der sich der Patient gut erholte.

Im Rahmen der elektiven Verlaufskontrolle im November 2022 präsentierte sich der Patient dem klinischen Vorzustand entsprechend mit einer milden Pallhypästhesie der unteren Extremitäten. Eine cMRT zeigte weiterhin keine neuen Läsionen, laborchemisch imponierte allerdings eine Leukozytopenie (2040 Zellen/µl) mit schwerer Neutropenie (100/µl), es waren keine CD20-positiven B‑Zellen im Blut nachweisbar.

Wir initiierten eine Therapie mit Filgrastim für 5 Tage, worunter es nach initial massivem Anstieg rasch wieder zu einem Abfall und im Anschluss zu einer anhaltenden Normalisierung der Granulozytenzahl kam (Abb. 1). Die Therapie mit OCR wurde beendet, eine Umstellung auf Ofatumumab unter engmaschigen Blutbildkontrollen ist geplant.

## Diskussion

Ocrelizumab (OCR) ist ein CD20-Antikörper, der zur Therapie der MS eingesetzt und als hoch wirksam eingestuft wird [5]. Fünf Jahre nach dessen Zulassung fehlen jedoch insbesondere im Hinblick auf seltenere therapieassoziierte Komplikationen noch Langzeitdaten [4]. Eine dieser seltenen, aber potenziell lebensbedrohlichen Komplikationen der Therapie mit CD20-Antikörpern stellen schwere Neutropenien (WHO-Grad 4) – mit einem Abfall der neutrophilen Granulozyten auf unter 500 Zellen/µl – wie oben beschrieben dar. Im klinischen Akutsetting müssen in solchen Fällen die wichtigsten Differenzialdiagnosen der Neutropenie, insbesondere Reaktionen auf andere Arzneimittel (z. B. auch Begleitmedikation im Rahmen der OCR-Gabe – in der Regel nichtsteroidale Antirheumatika, Antihistaminika und niedrigdosierte Glukokortikoide), Infektionen sowie eine insuffiziente Hämatopoese, bedacht werden.

Beide Kasuistiken fügen sich in eine Reihe von Fallberichten einer sog. Late-onset-Neutropenie (LON) mit Auftreten mehr als 4 Wochen nach OCR-Gabe ein, deren Mechanismus nicht abschließend geklärt ist. Ein ähnliches Phänomen ist allerdings von anderen CD20-Antikörpern, insbesondere Rituximab (RTX), bekannt. Diskutiert wird neben der Bildung antineutrophiler Antikörper sowie CD95-induzierter Apoptose durch große granulierte Lymphozyten (LGL) allen voran durch Dunleavy et al. ein alternativer zugrunde liegender Mechanismus [1, 7, 8]. Demnach sind im Rahmen der B‑Zell-Rekonstitution, die in der Regel etwa 3 Monate nach Rituximab-Gabe zu erwarten ist, hohe SDF-1-Spiegel („stromal cell-derived factor 1“, CXCL12) im Serum der Patienten nachweisbar, was eine Blockade des Ausreifens sowie der Migration neutrophiler Granulozyten aus dem Knochenmark ins Blut zur Folge haben könnte [1, 2].

Wie Rituximab wird auch OCR in 6‑Monats-Intervallen verabreicht, was eine ähnliche zeitliche Dynamik nahelegen könnte. Kommt es also zum Zeitpunkt der B‑Zell-Rekonstitution zu einem Granulozytennadir – unter RTX gemäß einer Übersichtsarbeit von Monaco et al. im Median an Tag 154 (± 87 Tage, Range 40–366 Tage; [6]), in den beiden beschriebenen Kasuistiken an Tag 66 bzw. Tag 177 – so birgt dies die Gefahr, durch die routinehaften Laborkontrollen nicht detektiert zu werden.

Eine Auswertung des FDA Adverse Event Reporting System (FAERS) identifizierte 25 Fälle einer LON unter OCR-Therapie (0,8 % der beobachteten Fälle; [3]). Das ermittelte Risiko liegt damit zwar deutlich unter dem des Auftretens einer LON bei RTX-Gabe (6,5 %; [6]); dies erscheint allerdings anhand der von RTX bekannten klinischen Inapparenz und der damit verbundenen hohen Dunkelziffer der LON sowie der schlechteren Datenlage zu OCR erklärbar [2, 6].

Ob es sich dabei zudem intraindividuell um Einzelfälle handelt oder ob es während der OCR-Therapie rezidivierend zu schweren Neutropenien kommen kann, ist unklar.

Unzureichend verstanden ist außerdem, welche Faktoren das Auftreten einer solchen Komplikation begünstigen. In oben genannter Auswertung des FAERS mit jedoch vergleichsweise geringer Fallzahl trat diese häufiger bei Patienten mit männlichem Geschlecht, höherem Alter und Untergewicht auf [3]. Inwieweit die vorausgegangene SARS-CoV-2-Infektion des zweiten Patienten relevant für das Auftreten der Lymphopenie war, bleibt ungeklärt.

Ein weiteres Dilemma stellt die Wahl der Anschlusstherapie dar. So ist völlig unklar inwieweit Patienten in Anbetracht des schwer zu beziffernden Rezidivrisikos einer LON von einer Therapieumstellung, wie in beiden beschriebenen Fällen geschehen, profitieren und inwieweit eine s.c. Applikation von CD20-Antikörpern (Ofatumumab, Fall 2) mit einem geringeren LON-Risiko assoziiert ist.

Letztlich bedarf es zum besseren Verständnis der Inzidenz, der zeitlichen Dynamik und prädestinierender Faktoren der LON unter OCR-Therapie sowie ggf. daraus resultierender therapeutischer Konsequenzen in Zukunft prospektiver Studien zu dieser klinisch äußerst relevanten Thematik.

## Fazit für die Praxis

- Die LON ist eine seltene, aber ernstzunehmende Komplikation der MS-Therapie mit CD20-Antikörpern.
- Durch ihren wahrscheinlich häufig asymptomatischen Verlauf und den Zeitpunkt des Auftretens ist denkbar, dass diese häufig durch das Raster der laborchemischen Routinekontrollen fällt. Ein Differenzialblutbild 3 Monate nach OCR-Gabe erscheint daher empfehlenswert.
- Die Wahl der Anschlusstherapie stellt eine Herausforderung dar.

## Interessenkonflikt

A. Berthele hat Honorare für Vorträge und Beratungen bekommen von Alexion, Biogen, Celgene, Novartis, Roche und Sandoz/Hexal. Seine Institution hat für die Teilnahme an klinischen Studien Kompensationen erhalten von Alexion, Biogen, Merck, Novartis, Sanofi und Roche. B. Hemmer ist in Scientific Advisory Boards für Novartis tätig. Er ist/war als DMSC Mitglied für AllergyCare, Polpharma, Sandoz, Biocom und TG therapeutics tätig. Seine Institution hat Forschungsmittel der Firma Regeneron und Roche für MS Forschung erhalten. Er ist an zwei Patenten mit Bezug zur MS beteiligt. F. Hess und P. Uibel geben an, dass kein Interessenkonflikt besteht.
